# What are the risk factors for avoidable transitions in the last year of life? A qualitative exploration of professionals’ perspectives for improving care in Germany

**DOI:** 10.1186/s12913-021-06138-4

**Published:** 2021-02-15

**Authors:** Alina Kasdorf, Gloria Dust, Vera Vennedey, Christian Rietz, Maria C. Polidori, Raymond Voltz, Julia Strupp, on behalf of CoRe Net, Christian Albus, Lena Ansmann, Frank Jessen, Ute Karbach, Ludwig Kuntz, Ingrid Schubert, Frank Schulz-Nieswandt, Stephanie Stock

**Affiliations:** 1 Department of Palliative Medicine, University of Cologne, Faculty of Medicine and University Hospital, Cologne, Germany; 2 Institute for Health Economics and Clinical Epidemiology, University of Cologne, Faculty of Medicine and University Hospital, Cologne, Germany; 3 Department of Educational Science and Mixed-Methods-Research, University of Education Heidelberg, Faculty of Educational and Social Sciences, Heidelberg, Germany; 4 Department II of Internal Medicine and Cologne Center for Molecular Medicine, Ageing Clinical Research, University of Cologne, Faculty of Medicine and University Hospital, Cologne, Germany; 5 Cluster of Excellence CECAD, University of Cologne, Cologne, Germany; 6 Center for Integrated Oncology Aachen Bonn Cologne Dusseldorf (CIO ABCD), University of Cologne, Faculty of Medicine and University Hospital, Cologne, Germany; 7 Clinical Trials Center (ZKS), University of Cologne, Faculty of Medicine and University Hospital, Cologne, Germany; 8 Center for Health Services Research, University of Cologne, Faculty of Medicine and University Hospital, Cologne, Germany

**Keywords:** Care transitions, Last year of life, Palliative care, Interview, Focus groups, Interprofessional perspectives

## Abstract

**Background:**

Little is known about the nature of patients’ transitions between healthcare settings in the last year of life (LYOL) in Germany. Patients often experience transitions between different healthcare settings, such as hospitals and long-term facilities including nursing homes and hospices. The perspective of healthcare professionals can therefore provide information on transitions in the LYOL that are avoidable from a medical perspective. This study aims to explore factors influencing avoidable transitions across healthcare settings in the LYOL and to disclose how these could be prevented.

**Methods:**

Two focus groups (*n* = 11) and five individual interviews were conducted with healthcare professionals working in hospitals, hospices and nursing services from Cologne, Germany. They were asked to share their observations about avoidable transitions in the LYOL. The data collection continued until the point of information power was reached and were audio recorded and analysed using qualitative content analysis.

**Results:**

Four factors for potentially avoidable transitions between care settings in the LYOL were identified: healthcare system, organization, healthcare professional, patient and relatives. According to the participants, the most relevant aspects that can aid in reducing unnecessary transitions include timely identification and communication of the LYOL; consideration of palliative care options; availability and accessibility of care services; and having a healthcare professional taking main responsibility for care planning.

**Conclusions:**

Preventing avoidable transitions by considering the multicomponent factors related to them not only immediately before death but also in the LYOL could help to provide more value-based care for patients and improving their quality of life.

**Supplementary Information:**

The online version contains supplementary material available at 10.1186/s12913-021-06138-4.

## Background

Patients in their last year of life (LYOL) represent a highly vulnerable group of patients. The care trajectory in the last months of life is characterized by multiple and often burdensome transitions, which are defined as a change between care settings, jeopardizing continuity of care [1]. Several studies found transitions increase in the last months of life [1–5], with most of them concerning hospitalization [1, 3, 4, 6–8]. The frequent hospital attendance at this stage of life contradicts most patients’ preference for home-based care [7, 9, 10]. The situation is similar in a longer period of the last 12 months of life [5, 6, 8, 11, 12]. The proportion of people who had been admitted at least once to hospital, nursing home or hospice in the LYOL ranged from 54% (France) to 76% (Austria, Israel, Slovenia) [11] while in Cologne (Germany) the value reached 91% [9]. In an Australian study nearly all of the decedents spent time in hospital with a marked increase in hospitalizations in the last 108 days of life for people who died of cancer [8]. According to a recent study of our group, the five most frequent transitions in the LYOL included hospital care. About half of them were from home to hospital, almost a quarter from hospital to home, and transitions between nursing home to hospital and hospital to hospital were less frequent [9]. Numerous transitions across healthcare settings in the last months of life are potentially avoidable [10, 13–16].

Most of these avoidable transitions are hospital admissions [14, 17–22]. In this context, terms such as unscheduled, avoidable, preventable, undesirable or inappropriate are used [20, 22, 23]. An inappropriate hospitalization can be avoided by better advance care planning or peer support for general practitioners (GPs) [24]. A clear-cut definition and classification is not available [24] and each individual situation needs to be evaluated to assess whether hopitalization is necessary. The predominant factor for admission to hospital are uncontrollable, such as acute medical situations occurring at home [25]. These transitions are initiated for curative or life-prolonging reasons [4]. Not all transitions are occurring for this reason: while effective symptom control, social and psychosomatic care can be mostly managed at home [26], an avoidable hospitalization takes place due to generalized weakness and social isolation rather than medical indication [14]. Another study specified avoidable hospitalization is due to insufficient nursing provision or a lack of family support [21]. System factors (poor availability of alternative sites of care or failure of preventive actions by other healthcare professionals (HCPs)), social and family factors were identified as drivers for hospitalization in the last month of life of older adults [24]. It is unclear to what extent these risk factors for transitions are applicable to the German context, which of the factors are considered avoidable by German HCPs and what suggestions HCPs have for overcoming these transitions. To close this gap, this paper focuses on such avoidable transitions in the LYOL.

## Methods

### Study design

A qualitative study with two focus groups (FG) and five interviews was conducted. To better understand avoidable transitions in the LYOL, we explored perspectives from HCPs. This article reports data from a larger mixed-methods cross-sectional study, which was undertaken to study the LYOL in Cologne [27]. The study was prospectively registered on 13th June 2017 in the German Clinical Trials Register (DRKS00011925).

### Participants

The data were collected between May and October 2019. Participants were a purposive sample of hospital physicians, general practitioners (GPs), nursing staff (NS), outpatient specialists and members from hospice, outpatient palliative care teams and care homes, with different qualifications and experience in providing healthcare for patients in their LYOL. All potential participants received invitations and an information sheet per email from a member of the research team to encourage participation. A snowball sampling-technique was used, supported by our field access partners. Face-to-face-interviews were conducted with nurses and GPs who wanted to participate in the FG but were unable to attend them. The FGs were designed homogeneously in terms of function - in order to avoid hierarchy biases - and heterogeneously in terms of occupational field and pool of experience. The setting was designed to make respondents feeling comfortable mentioning other professions without making themselves vulnerable or offending others.

### Data collection

Data were collected by research associates GD, VV and AK, all of whom have a master degree and previous experience in qualitative data collection. They were unknown to all study participants bar one at the time of data collection. Both focus group and interview participants received the same questions to ensure comparability of the data. A semi-structured interview guideline was developed using relevant literature [6, 17, 20–22, 28]. The guideline covered several key areas regarding risk factors for avoidable transitions between care settings in the LYOL and included the same questions for both the FGs and the interviews (Supplemental file 1). Demographic data of participants was collected. Participants provided written informed consent and the study was approved by the Ethics Committee of the Medical Faculty of the University of Cologne (#17–188). Respondents also received an incentive for their participation (€50). The FGs and three interviews were held at the University Hospital Cologne in the time between the day and afternoon shifts. Two interviewees preferred being interviewed at their place of work. Data Collection was continued until the point of information power was achieved [29].

### Data analysis

The qualitative data were audio-recorded, transcribed verbatim and then coded by the first author (AK). The thematic framework was modified in consensus with two further researchers (GD & VV). Transcripts were analysed with MAXQDA [30] using Miles and Huberman’s guidelines for qualitative content analysis [31]. Due to the explanatory character of our study, the coding scheme was deductively based on available research evidence [6, 12, 17, 32] and inductively derived from the content of the interview transcripts. All places, names and identifiable information were anonymized during transcription.

## Results

### Participant characteristics

Two FGs were formed involving specialist physicians (SP) (*n* = 3) and managers (*n* = 8) from different disciplines. Not all participants who had agreed to participate in a focus groups attended, from a total of 13 participation commitments 11 came to the FG. FGs lasted for approximately 110 min and the individual interviews lasted between 31 and 69 min (average: 52 min). Three GPs and two hospital nurses participated in the study in face-to-face interviews. In total, 16 HCPs from generalist and specialist palliative care settings participated.

Table 1 summarizes the characteristics of HCPs. The specialist physicians were specialized in anaesthesiology, geriatrics, intensive, emergency, palliative and internal medicine and worked in a hospital setting. The managers were defined as staff in executive positions with human resources responsibility and decision-making authority in hospice, care home, hospital, outpatient or nursing service.

**Table 1:** Characteristics of healthcare professionals (*n* = 16)

	Specialist physician	Manager	General practitioner^a^	Nursing staff
N	3	8	3	2
Mean age	46.3	47.4	57.7	38
Sex (female; %)	2	4	1	1
Specialist PC^b^	1	3	–	–
**Work experience**				
less than 5 years	1	1	–	–
5–10 years	1	2	–	1
11–20 years	–	1	1	1
more than 20 years	1	4	2	–

^a^individual interviews ^b^palliative care

### Potentially risk factors for avoidable transitions in the LYOL

**Fig. 1:**
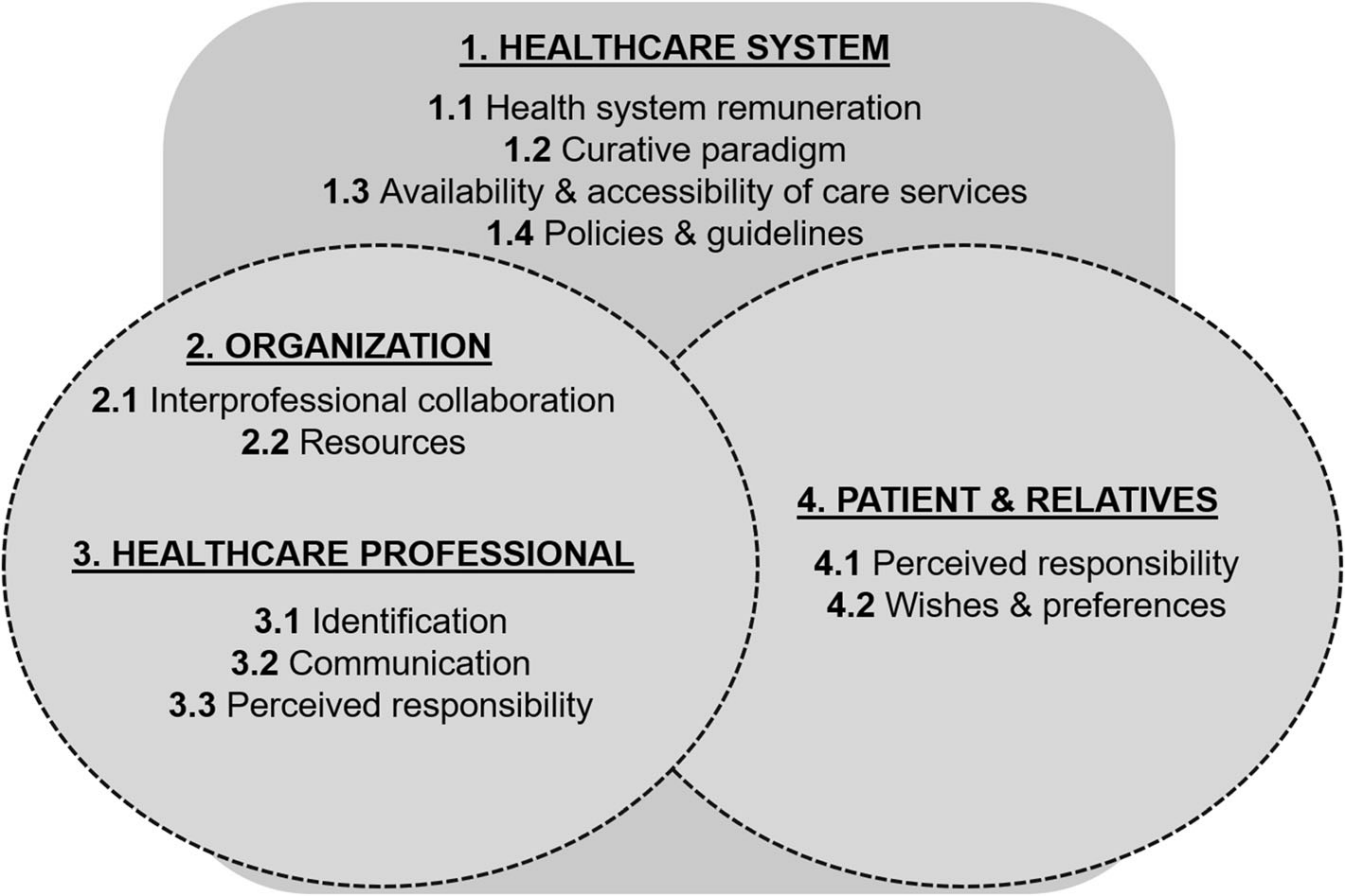
Potentially risk factors for avoidable transitions in the LYOL)

Figure 1 summarizes the potential risk factors for transitions in the LYOL, which are described in detail below. Risk factors for avoidable transitions in the LYOL could be clustered into four groups: health system, organization, HCP, patient and relatives. The participants were able to detect a strong interrelation between these four factor groups. Each of these four themes seem to have sub-themes, as shown in the figure.

### Healthcare system as factor

#### Health system remuneration

The respondents perceived a high frequency of economic orientation of care services, especially in hospital settings. One physician suggested hospitals rely primarily on diagnostic methods to increase revenues, generating avoidable transitions in the LYOL (SP#137)

So, in principle, we have the problem that hospitals rely on diagnostic tools with which they can make relatively large sums of money [ … ]. (SP#136)

Simultaneously, the participants emphasized a lack of remuneration for “spoken” medicine, meaning general medical consultation on therapy options.

#### Curative paradigm

According to the participants, there is a prevailing curative paradigm of healthcare delivery. Care decisions are often based on curative treatment without considering palliative care approaches, generating avoidable transitions.

It’s a lot more difficult to just do nothing instead of giving standard [curative] treatment, but you have to remember that nothing doesn’t mean nothing. I have to fill the “nothing” with specific things and actions. So “nothing” actually only means “not the hospital”. (GP7#200)

One nurse stated that the treatment strategy for hospital patients is oriented towards a reaction to their acute complaints and less towards a long-term view of the disease in a holistic context.

There are only reactions [in the form of treatment] to the [patient’s] acute situation, and then another reaction, and another reaction. (NS5#177)

#### Availability and accessibility of care services

Participants complained about limited capacity of healthcare settings such as hospices or short-term care. One participant described the difficulty of finding a nursing service after being discharging from hospital. Additionally, the participants do not feel sufficiently informed about the existing palliative care services. An avoidable transition to hospital can also occur if a bed in a hospice or outpatient home care is unavailable. A physician listed the reasons for an avoidable hospitalization:

For example, that the family [...] is simply overwhelmed by the situations that arise, even if the patient has the explicit wish to die at home. Well, then either the care has not yet been started because, for example, they have registered for specialized outpatient palliative care, but the service has not yet begun, or they have registered for placement in a hospice, but they are still waiting for placement, or there are symptom crises that cannot be managed because the family can’t reach the specialized outpatient palliative care or the specialist palliative physician [...]. (SP#54)

Among the specific suggestions to prevent unnecessary transitions participants mentioned an around-the-clock outpatient emergency telephone hotline in the event of acute symptom crises.

#### Policies & guidelines

The German healthcare system is extremely fragmented and is governed by strict legal regulations. Problems often arise at the interfaces between inpatient and outpatient care. One GP described an inadequate implementation of discharge management in hospitals as a consequence of such regulations:

There are hospital discharges that are, they're not well-prepared. There are discharges that are made on Fridays around noon. And nothing has been organized [for the patient]. The flow of information is seldom good, at least for me [involved GP]. Then I don't even find out [that my patient has been discharged]. (GP#135)

Respondents complained that communication with other HCPs is made more difficult by restrictive data protection. According to the participants, data protection regulations sometimes prohibit physicians from contact hospices without patient consent. Especially in by discharging patients from a hospital, complications from consequent discharge management were reported:

When patients go from a hospital ward to short-term care, the short-term care is at the site [hospital], but legally belongs to the outpatient area. This means that I cannot go up there and prescribe anything. This should actually be done by the practitioner in a private practice, or by the specialist palliative home care team [ … ]. (SP#58)

### Organization as factor

#### Interprofessional collaboration

The cooperation with other HCPs, which is partly characterized by limited out-of-hours service availability, the use of different communication media and a lack of contact persons was described as possible factors for avoidable transitions in the LYOL.

Everybody complains about other colleagues, right? We at the nursing home can also say: “Boy, what have they done in the hospital *this* time? And why is it that way?” Maybe they think the same thing about us. (manager#197)

Respondents reported informal arrangements with other HCPs to maintain the continuity of care, preventing out-of-hours service hospitalization, and entering an “alliance”. One GP described individual temporary solutions arising within the framework of an agreement with the outpatient nursing services and relatives:

[...] I have actually always tried to make a deal with a nursing home, a nursing service, or with the relatives: “If something goes wrong, call me, hm. But I also have to be willing to give out a mobile phone number where I can be reached after hours”. (GP#144)

#### Resources

According to the respondents, the role of GPs is to assess the domestic situation of the affected person or their acute state of health in order to formulate an adequate care indication plan to avoid redundant hospitalizations in the LYOL. However, this kind of medical assessment is hindered due to the limited time for home visits.^1^ Care homes and nursing services are particularly affected by resource restrictions and are therefore often criticized by the participants. They also reported a general shortage of resources, one manager emphasized:

The pressures that we are all under here in the healthcare system are often that of extreme time pressure and staff pressure. It doesn't matter whether it's in the outpatient or inpatient facility, it’s in the hospital. (manager#197)

If curative treatments continue for a considerable part of the illness trajectory, the respondents suggested a lack of competence of the responsible HCP. Palliative care competence was identified as a crucial but predominantly missing resource for the identification of patients in their LYOL and effective management of symptom crises. It seems to affect young physicians in particular. Participants perceived a lack of palliative care competences among medical professionals including the application of sedative drugs:

Then it’s really easy [for others] to say, “Then inject some morphine.” No one has a problem with insulin. But when you have the morphine injection in your hand it's the fear of killing [the patient], yes, that something might happen. (manager#74)

### Healthcare professionals as factor

#### Identification

The reasons for the persistence of curative treatment and late identification of the LYOL were attributed to different factors. Respondents mentioned the lacking competence of the HCP to identify a LYOL-patient. Besides, it was assumed the HCPs are unwilling to take a closer look at the patient’s health status. Furthermore, a lack of standardized methods for identifying patients in their LYOL was assumed.

The nurses most often address the assistants or the senior physicians to talk to the patients, because they [nurses] have more contact with them. They also have a little more sensitivity and say, ‘This is actually a patient who can be palliative, [ … ] in other words, after the question, “Would it surprise you if this patient does not survive the next six to twelve months?” SP#25

#### Communication

Respondents stated that an honest and early communication about entering the LYOL is necessary but often lacking for patients. One nurse complained that empathetic communication and information for patients before treatment are rare and emphasized the need for more patience and interest on the part of HCPs. The timing for the breaking bad news (BBN) was highlighted:

And I think several things can be avoided if everything is carefully considered at an early stage and not only when everything is already absolutely palliative anyway. (manager#178)

The perceived health condition of the patients is largely dependent on whether, and how well, the information about the state of health has been provided:

[I] t is partly due to the patients saying: “No, I want to continue treatment”. So sometimes I think that the patients are not sufficiently informed about the actual state of their illness. (NS5#25)

The aspect of BBN was associated with anticipatory discussions about the care planning, preventing avoidable transitions. They indicated the issue of lacking communication about the prospect of death, attributed to various reasons such as the fear of discussing death or the lacking time. Additionally, the inexperience of assistant physicians was mentioned orienting towards diagnostic processes rather than communicative aspects of treatment goals.

#### Perceived responsibility

According to the respondents, a certain degree of assuming or taking on responsibility is necessary for the implementation of BBN. A role as “gatekeeper” was consensually assigned to the GPs. If one HCP does not take on a central role in the care provision, there is a clear shift in responsibility towards other HCPs, creating a diffusion of responsibility. This circumstance could be disadvantageous to the patients in their LYOL.

[ … ] There is no one who ultimately has any vision. Yes. And that's, that-- that's one of the main problems in the whole clinical process. (SP#117).

The ability to assume responsibility for the patients in their LYOL was stated as closely linked to the personality of the HCP:

It can't be done without the commitment of healthcare professionals, yes. The commitment must be there. [...] People always say it's about money. I do not believe it. I think it is really something that appeals very much to character traits or, yes, I would say, also to the meaning of my work, yes. I'm not a counter clerk who hands out stamps, I have a care contract with the patients. That doesn't mean I have to sit on the side of their bed every day. But I do have a clear agenda, and I stand behind it. (GP4#150)

### Patient and relatives as a factor

#### Perceived responsibility

The respondents found relatives are mainly responsible for the care of patients in their LYOL. Extreme examples were reported of relatives taking patients to hospital avoiding dealing with a problem themselves, which was referred to as “practice of shuffling off responsibility”.

Nevertheless, the attitude [of some relatives] is: “Well, I can't adjust my life because she's [patient] no longer cared for. And you can't discharge her with insufficient care, so she will have to stay here, you have to take care of her.” And sometimes people stay in hospital for weeks until a short-term care bed is available somewhere. And I don't even want to ask, yes, but it would be interesting to find out what is really going on there. Basically, we just push off the problem to short-term care because that is limited. And they will have the same difficulties with the long-term care, won't they? (SP#213)

#### Wishes & preferences

Respondents reported difficulties in dealing with family wishes that conflicted with those of the patients.

[ … ] I mean, there are of course some patients who come in such desolate condition that they are not really responsive, are confused and so on. But even then, there are the relatives involved who say: “we are just taking him to hospital again”. (NS5#65)

Participants explained hospitalizations during the LYOL happen despite the patients and relatives having adequate information and/or adequate home care. The emergence of such transitions was associated as the result of growing emotional, physical, and financial stress that the caregiver must bear during this time.

## Discussion

Avoidable transitions in the last year of life are an obstacle for the continuity of care for patients. This article highlights aspects of avoidable transitions from the perspective of HCP, in an extended 12-months timeframe. As already observed in a study involving 15 European countries, patients are affected by many transitions in their LYOL, which cannot only be explained by medical needs [11]. The risk factors for avoidable transitions identified in this study are similar to those already identified in the literature. The identified risk factors are classified into four groups: healthcare system, organization, HCP, patient and relatives (Fig. 1). These results reveal the complexity of the factors influencing avoidable transitions between healthcare settings.

From the perspective of HCP, the characteristics of the healthcare system pose a risk for the occurrence of avoidable transitions that require improvement. An additional demand includes consistent discharge management, a more flexible approach to data protection and the use of modern technologies, especially in hospitals. Similar to our results, the availability and accessibility of receiving community services were identified as a potential risk factor for a hospitalization also near the final stage of life [6, 10, 20, 22, 23, 34]. Both overcoming curative thinking towards openness to palliative care and structural reform of the health system are needed to avoid the strong economic focus of health organizations on diagnostic and curative treatment in the LYOL. The predominant curative thinking of HCP was often mentioned by respondents as a ‘rescue culture’ and was already been mentioned in a New Zealand study [20]. Consistent with our results, a Dutch study found that marking the approach of death, and shifting the mindset could help to avoid hospitalization at the end of life [17]. Current issues of transitions in the LYOL are revealed to the communication complexities, care planning, coordination gaps and health system reform needs [12]. The identified risk factors of the healthcare system for avoidable transitions are in line with findings in other studies, therefore it can be assumed that these are not based on the characteristics of the German healthcare system alone, regarding the sector boundary between outpatient, inpatient care and rehabilitation facilities [35].

Risk factors were also identified at the organizational level. More effective interprofessional collaboration and communication between HCPs and patients may constitute effective tools for preventing avoidable transitions of patients in their LYOL. Overall, there is an explicit need for an expansion of palliative care qualifications (both medical and nursing staff, young medical staff in particular), around-the-clock outpatient emergency telephone hotline, expansion of organizational resources and centralized information on the availability of adequate care. While palliative care is often perceived to be indicated typically for dying people and those with major pain issues, the benefits and fields of applications of palliative care are considered much wider nowadays. Palliative care contributes to symptom management, family support, coping strategies etc. in the care for various diseases [36, 37]. Therefore, early involvement of palliative care practitioners can be helpful for managing various symptoms next to pain and regardless of the anticipated remaining life span [36].

HCPs have identified themselves as a risk factor for the emergence of avoidable transitions. The perception of own responsibility for the patients is representing a feature that could stimulate the prevention of avoidable transitions. Consistent with existing literature, GPs play a pivotal role in this context, ensuring the continuity of care and taking overall responsibility [38, 39].

Interventions for focused support of caring relatives are desirable to avoid a “practice of shuffling off responsibility” by the relatives, defined as distributing one’s own responsibility to others. The results of our study show that timely identification and anticipatory discussions about the current health status and care planning in the LYOL could build the foundation of efficient care provision in the context of avoidable transitions. Anticipatory discussions and interventions to deal with expected severe problems could lower the frequency of avoidable transitions as already stated in the literature [10, 17, 34, 40]. However, if the patients are not identified, such discussions cannot be held. And the identification of LYOL patients remains difficult. The existing communication problems between patients and HCPs seem to be present regardless of the time before death [12, 22, 28, 41] which emphasizes the urgency of having such discussions as early as possible.

Patient and relatives as a factor were also considered as potential risk factors for avoidable transitions in the LYOL. Greater consideration and information for both patients and their relatives to prevent avoidable transitions in the LYOL may be achieved through the remuneration of “spoken” medicine, helping the HCPs to hold such conversations. To address the assumption that consultations with patients are not conducted because they are not remunerated, an adjustment in the payment system is desirable. Additionally, the minimally invasive methods such as surprise question could be an adequate way to identify such patients [42].

This paper emphasizes the importance of consideration of avoidable transitions in the context of the last 12 months of life, not only shortly before death. Several studies are mostly concentrated on the last 6 months [3, 26], last 3 months [2, 4, 17, 40, 43, 44], last 30 days [1, 13, 45] or last 72 h [22, 46]. The other risk factors for avoidable transitions identified in the literature also appear to be independent of time. However, the consideration of the few months could be regarded as shortened, since the transitions can be avoided much earlier. The benefits of an extended period of 1 year for considering avoidable transitions are obvious, especially since minimally invasive techniques for identifying patients in the LYOL such as surprise question already existing (“Would you be surprised if the patient were to die in the next 12 month?”) [42]. Although the predictive value of the surprise question is poor to modest [47], it could be sufficient for a subliminal consideration of an indication for palliative care. The surprise question is a better alternative to intuition, being a valuable tool to facilitate consideration of the palliative care needs [48, 49].

The results of this study allow the following considerations. Regarding the aspect of avoidable transitions in an extended period of 12 months could help provide new perspectives on healthcare provision for patients in their LYOL. The early identification of entering the LYOL can reduce avoidable transitions through appropriate healthcare planning, which is both beneficial for patient-oriented care and the economic context. Thus, interventions to reduce or modify avoidable transitions should be taken already as part of the overall care of patients with life-threatening diseases. In this context, comprehensive information about the progression of the disease as well as care planning for patients and their relatives plays a central role by preventing avoidable transitions [50]. But still there are difficulties in identifying and communicating the entry into the LYOL.

### Strengths and limitations

Best of our knowledge, there is no multidisciplinary analysis of the risk factors for avoidable transitions between care settings in the LYOL in Germany. However, some limitations must be considered. In comparison, the specific features of each healthcare system need to be considered, since some results are not transferable to healthcare systems with different structures or be generalized for the rural context. As a possible confounding factor, only HCP with an interest in the subject of care have volunteered to participate. Due to recruiting difficulties for a FGs, the GPs and NS were only interested in face-to-face interviews. Therefore, the results could be different if they had been conducted in the presence of these professionals. Another limitation of the study is that we did not include social workers or case managers in our sample. The perspective of these providers on the presentation of avoidable excesses is therefore missing. The use of the focus group discussion method is subject to its own limitations [51], while the overall sample of the study can be considered small but purposive.

## Conclusion

Based on the results of this study, it is recommended to consider the aspect of avoidable transitions in an extended period of 12 months and not only shortly before death. Four groups of risk factors need to be considered: healthcare system, organization, HCP, patient and relatives. The most persistent problem is the lack of or late identification of patients in the LYOL. A treatment trajectory free of avoidable transitions can only be designated after a person has been identified as a LYOL-patient. Since most avoidable transitions result from lacking identification of the patient’s LYOL, the use of the identification tools could be an effective method to optimize the current provision practice.

## Supplementary Information

**Additional file 1: Supplemental file 1.** Focus group/ interview guideline.

## Data Availability

The datasets used and/or analysed during the current study are available from the corresponding author on reasonable request.

## Abbreviations

FG: Focus group
GP: General practitioner
HCP: Health care professional
LYOL: Last year of life
NS: Nursing staff
SP: Specialist physicians
CoRe-Net: Cologne-Research-and-Development-Network

## Footnotes

1 Home visits are an integral part of general practice in Germany and predominantly delivered by family practitioners working solo or in small practices. However, over recent years the frequency of home visits has decreased [33].

## References

[1] Abraham S, Menec V (2016). Transitions between care settings at the end of life among older homecare recipients: a population-based study. Gerontol Geriatr Med.

[2] Abarshi E, Echteld M, van den Block L, Donker G, Deliens L, Onwuteaka-Philipsen B (2010). Transitions between care settings at the end of life in the Netherlands: results from a nationwide study. Palliat Med.

[3] Baehler C, Signorell A, Reich O (2016). Health care utilisation and transitions between health care settings in the last 6 months of life in Switzerland. Plos One.

[4] van den Block L, Pivodic L, Pardon K, Donker G, Miccinesi G, Moreels S (2015). Transitions between health care settings in the final three months of life in four EU countries. Eur J Pub Health.

[5] Ko W, Deliens L, Miccinesi G, Giusti F, Moreels S, Donker GA (2014). Care provided and care setting transitions in the last three months of life of cancer patients: a nationwide monitoring study in four European countries. BMC Cancer.

[6] Hanratty B, Lowson E, Grande G, Payne S, Addington-Hall J, Valtorta N et al. Transitions at the end of life for older adults – patient, carer and professional perspectives: a mixed-methods study. Southampton; NIHR Journals Library; Health Services and Delivery Research 2.17, 2014. 10.3310/hsdr02170. 25642566

[7] Bone AE, Gao W, Gomes B, Sleeman KE, Maddocks M, Wright J (2016). Factors associated with transition from community settings to hospital as place of death for adults aged 75 and older: a population-based mortality follow-Back survey. J Am Geriatr Soc.

[8] Rosenwax LK, McNamara BA, Murray K, McCabe RJ, Aoun SM, Currow DC (2011). Hospital and emergency department use in the last year of life: a baseline for future modifications to end-of-life care. Med J Aust.

[9] Voltz R, Dust G, Schippel N, Hamacher S, Payne S, Scholten N (2020). Improving regional care in the last year of life by setting up a pragmatic evidence-based plan–do–study–act cycle: results from a cross-sectional survey. BMJ Open.

[10] Giezendanner S, Bally K, Haller DM, Jung C, Otte IC, Banderet H-R (2018). Reasons for and frequency of end-of-life hospital admissions: general Practitioners’ perspective on reducing end-of-life hospital referrals. J Palliat Med.

[11] Overbeek A, van den Block L, Korfage IJ, Penders YWH, van der Heide A, Rietjens JAC (2017). Admissions to inpatient care facilities in the last year of life of community-dwelling older people in Europe. Eur J Pub Health.

[12] Wilson DM, Birch S (2018). Moving from place to place in the last year of life: a qualitative study identifying care setting transition issues and solutions in Ontario. Health Soc Care Community.

[13] Allers K, Hoffmann F, Schnakenberg R (2019). Hospitalizations of nursing home residents at the end of life: a systematic review. Palliat Med.

[14] Cornillon P, Loiseau S, Aublet-Cuvelier B, Guastella V (2016). Reasons for transferral to emergency departments of terminally ill patients - a French descriptive and retrospective study. BMC Palliat Care.

[15] Walsh EG, Wiener JM, Haber S, Bragg A, Freiman M, Ouslander JG (2012). Potentially avoidable hospitalizations of dually eligible Medicare and Medicaid beneficiaries from nursing facility and home- and community-based services waiver programs. J Am Geriatr Soc.

[16] Ouslander JG, Maslow K (2012). Geriatrics and the triple aim: defining preventable hospitalizations in the long-term care population. J Am Geriatr Soc.

[17] de Korte-Verhoef MC, Pasman HRW, Schweitzer BPM, Francke AL, Onwuteaka-Philipsen BD, Deliens L (2015). How could hospitalisations at the end of life have been avoided? A qualitative retrospective study of the perspectives of general practitioners, nurses and family carers. PLoS One.

[18] Donzé J, Lipsitz S, Schnipper JL (2014). Risk factors for potentially avoidable readmissions due to end-of-life care issues. J Hosp Med.

[19] Gott M, Gardiner C, Ingleton C, Cobb M, Noble B, Bennett MI (2013). What is the extent of potentially avoidable admissions amongst hospital inpatients with palliative care needs?. BMC Palliat Care.

[20] Gott M, Frey R, Robinson J, Boyd M, O'Callaghan A, Richards N (2013). The nature of, and reasons for, ‘inappropriate’ hospitalisations among patients with palliative care needs: a qualitative exploration of the views of generalist palliative care providers. Palliat Med.

[21] Hoare S, Kelly MP, Barclay S (2019). Home care and end-of-life hospital admissions: a retrospective interview study in English primary and secondary care. Br J Gen Pract.

[22] Hoare S, Kelly MP, Prothero L, Barclay S (2018). Ambulance staff and end-of-life hospital admissions: a qualitative interview study. Palliat Med.

[23] Ouslander JG, Lamb G, Perloe M, Givens JH, Kluge L, Rutland T (2010). Potentially avoidable hospitalizations of nursing home residents: frequency, causes, and costs. J Am Geriatr Soc.

[24] Cardona-Morrell M, Kim JCH, Brabrand M, Gallego-Luxan B, Hillman K (2017). What is inappropriate hospital use for elderly people near the end of life? A systematic review. Eur J Intern Med.

[25] Reyniers T, Houttekier D, Cohen J, Pasman HR, Deliens L (2014). The acute hospital setting as a place of death and final care: a qualitative study on perspectives of family physicians, nurses and family carers. Health Place.

[26] Barbera L, Taylor C, Dudgeon D (2010). Why do patients with cancer visit the emergency department near the end of life?. CMAJ.

[27] Strupp J, Hanke G, Schippel N, Pfaff H, Karbach U, Rietz C (2018). Last year of life study Cologne (LYOL-C): protocol for a cross-sectional mixed methods study to examine care trajectories and transitions in the last year of life until death. BMJ Open.

[28] McDermott C, Coppin R, Little P, Leydon G (2012). Hospital admissions from nursing homes: a qualitative study of GP decision making. Br J Gen Pract.

[29] Malterud K, Siersma VD, Guassora AD (2016). Sample size in qualitative interview studies: guided by information power. Qual Health Res.

[30] VERBI Software: MAXQDA, software for qualitative data analysis. Berlin; 2019. Available from: URL: maxqda.com. [cited 2020 Aug 15]

[31] Miles MB, Huberman AM, Saldaña J (2014). Qualitative data analysis: a methods sourcebook. Edition 3.

[32] Gott M, Ingleton C, Gardiner C, Richards N, Cobb M, Ryan T et al. Transitions to palliative care for older people in acute hospitals: a mixed-methods study. Southampton; NIHR Journals Library; Health Services and Delivery Research 1.11; 2013. 10.3310/hsdr01110. 25642530

[33] Pochert M, Voigt K, Bortz M, Sattler A, Schübel J, Bergmann A (2019). The workload for home visits by German family practitioners: an analysis of regional variation in a cross-sectional study. BMC Fam Pract.

[34] Ong ACL, Sabanathan K, Potter JF, Myint PK (2011). High mortality of older patients admitted to hospital from care homes and insight into potential interventions to reduce hospital admissions from care homes: the Norfolk experience. Arch Gerontol Geriatr.

[35] Institute for Quality and Efficiency in Health Care. Health care in Germany: The German health care system. In: InformedHealth.org; 2018. Available from: URL: https://www.ncbi.nlm.nih.gov/books/NBK298834/. [cited 2021 Jan 15]

[36] Temel JS, Greer JA, Muzikansky A, Gallagher ER, Admane S, Jackson VA (2010). Early palliative care for patients with metastatic non-small-cell lung cancer. N Engl J Med.

[37] Radbruch L, de Lima L, Knaul F, Wenk R, Ali Z, Bhatnaghar S (2020). Redefining palliative care-a new consensus-based definition. J Pain Symptom Manag.

[38] Michiels E, Deschepper R, van der Kelen G, Bernheim JL, Mortier F, Vander Stichele R (2007). The role of general practitioners in continuity of care at the end of life: a qualitative study of terminally ill patients and their next of kin. Palliat Med.

[39] Reyniers T, Houttekier D, Pasman HR, Stichele RV, Cohen J, Deliens L (2014). The family Physician's perceived role in preventing and guiding hospital admissions at the end of life: a focus group study. Ann Fam Med.

[40] de Korte-Verhoef MC, Pasman HRW, Schweitzer BPM, Francke AL, Onwuteaka-Philipsen BD, Deliens L (2014). Reasons for hospitalisation at the end of life: differences between cancer and non-cancer patients. Support Care Cancer.

[41] Reyniers T, Deliens L, Pasman HRW, Vander Stichele R, Sijnave B, Houttekier D (2017). Appropriateness and avoidability of terminal hospital admissions: results of a survey among family physicians. Palliat Med.

[42] White N, Kupeli N, Vickerstaff V, Stone P (2017). How accurate is the ‘Surprise Question’ at identifying patients at the end of life? A systematic review and meta-analysis. BMC Med.

[43] de Korte-Verhoef MC, Pasman HRW, Schweitzer BP, Francke AL, Onwuteaka-Philipsen BD, Deliens L (2014). General practitioners’ perspectives on the avoidability of hospitalizations at the end of life: a mixed-method study. Palliat Med.

[44] Pivodic L, Pardon K, Miccinesi G, Vega Alonso T, Moreels S, Donker GA (2016). Hospitalisations at the end of life in four European countries: a population-based study via epidemiological surveillance networks. J Epidemiol Community Health.

[45] Gozalo P, Teno JM, Mitchell SL, Skinner J, Bynum J, Tyler D (2011). End-of-life transitions among nursing home residents with cognitive issues. N Engl J Med.

[46] Morris ZS, Fyfe M, Momen N, Hoare S, Barclay S (2013). Understanding hospital admissions close to the end of life (ACE) study. BMC Health Serv Res.

[47] Downar J, Goldman R, Pinto R, Englesakis M, Adhikari NKJ (2017). The “surprise question” for predicting death in seriously ill patients: a systematic review and meta-analysis. CMAJ.

[48] Gerlach C, Goebel S, Weber S, Weber M, Sleeman KE (2019). Space for intuition - the ‘Surprise’-question in haemato-oncology: qualitative analysis of experiences and perceptions of haemato-oncologists. Palliat Med.

[49] Haydar SA, Almeder L, Michalakes L, Han PKJ, Strout TD (2017). Using the surprise question to identify those with unmet palliative care needs in emergency and inpatient settings: what do clinicians think?. J Palliat Med.

[50] Polak L, Hopkins S, Barclay S, Hoare S (2020). The difference an end-of-life diagnosis makes: qualitative interviews with providers of community health care for frail older people. Br J Gen Pract.

[51] Smithson J (2000). Using and analysing focus groups: limitations and possibilities. Int J Soc Res Methodol.

