# Supplementary material for: What are the risk factors for avoidable transitions in the last year of life? A qualitative exploration of professionals’ perspectives for improving care in Germany
Source: BMC Health Serv Res. 2021 Feb 15;21:147. doi: 10.1186/s12913-021-06138-4 (PMC7885553; doi:10.1186/s12913-021-06138-4)
Supplement: Supplementary file 1 — Additional file 1: Supplemental file 1. Focus group/ interview guideline. [file 12913_2021_6138_MOESM1_ESM.docx]

Focus group/interview guideline

- Introduction
- Answer any questions participants have
- Explain the purpose of the focus groups/interview
- Take consent
- Distribution of demographic data

*Section I: Patients in their last year of life*

1. Please describe us how you experience patients in their last year of life.
   1. Special characteristics? Differences between these patients? Are there special features in pain and symptom management?
   2. How can we recognize that the patient is in the last year of life? Are there (standardized) procedures for the identification? Possibilities of the identification in the hospital?

*Section II: Transitions in the last year of life*

[a chart with increasing number of transitions in the last year of life is shown, based on the previous project results]

1. What do you think about increasing transitions in the last year of life?
   1. What triggers can you identify that are related to the transitions in the last year of life? How do the transitions occur?
   2. Which transitions are necessary? Who decides on the initiation of these transitions? Who communicates this, how and to whom? Who is responsible?
   3. How can transitions in the last year of life succeed? What problems can arise? What would be necessary to improve cooperation with another stakeholder?
2. What could you personally do to make the transitions in the last year of life different?
3. Is there anything else on the topic of transitions in the last year of life that has not yet been discussed, but you would like to conclude?
